# Is sustainable marketing based on virtue ethics the answer to addressing socio-ecological challenges facing humankind?

**DOI:** 10.1007/s13162-021-00200-4

**Published:** 2021-05-28

**Authors:** Andrea Prothero, Pierre McDonagh

**Affiliations:** 1 UCD Centre for Business and Society (CeBaS), University College Dublin, Dublin, Ireland; 2 Marketing, Business and Society Division, School of Management, University of Bath, Bath, UK

**Keywords:** Sustainability, Virtue ethics, Marketing mix paradigm, Marketing 4Ps, Ecofeminism

## Abstract

This commentary provides a review of Dyck and Manchanda’s work on the use of virtue ethics, through Sociological and Ecological Thought (SET) Oriented Marketing, in tackling socio-ecological challenges within society. While we concur with the focus of the paper on moving away from a central emphasis on profit maximization, we differ in how we believe this can be achieved. We critique the SET approach put forward from three key positions: (a) the SET approach and the application of virtue ethics; (b) the SET approach and the use of the marketing mix to operationalize it in practice; and, (c) the missing systemic and institutional barriers which we believe render SET problematic both theoretically and in practical terms. We conclude by suggesting that instead of utilising normative ethical theories to address socio-ecological challenges marketing researchers turn to other perspectives, such as ecofeminism.

We would like to begin by applauding Dyck and Manchanda (2021) for their thoughtful and insightful article which explores how virtue ethics can be applied in a marketing context to tackle the socio-ecological challenges facing humankind. They do so through what they have labelled Social and Ecological Thought (SET) Oriented Marketing. As the authors rightly point out, for many decades now there has been considerable critique of marketing’s role in the climate crisis and ecological degradation faced by the world (Fisk, 1973; Kilbourne et al., 1997; Wilkie & Moore, 2012) alongside broader critiques of business for profit maximization at the expense of more pressing social and ecological issues (Banerjee, 2003; Elkington, 1997; Shrivastava, 1995).

While not offering a definition of SET per se, the authors emphasize it is a thought process based on the use of virtue ethics which “aims to optimize social and ecological well-being while ensuring financial viability” (p 1). The authors contend SET recognises that a focus on profit maximization is unethical and instead suggest firms should prioritize “flourishing community ahead of firm’s and individuals financial well-being” (p 3). From a marketing context, the authors take marketing’s 4Ps (product, price, place, promotion) as a means through which to apply their SET approach. Such an approach can be criticised by many who argue that the “business of business is business” (Friedman, 1962) and that profit maximization is key, as per the utilitarian arguments put forth by the authors.

For our purposes, we concur with the authors that a primary focus on profit maximization is of great concern from both social and ecological perspective. Where we differ however is in *how* to address these concerns. Below we offer a critique of the SET approach proffered by Dyck and Manchanda, from theoretical and practical perspectives. Specifically we focus on: (a) the SET approach and the application of virtue ethics; (b) the use of the marketing mix; and (c) the missing systemic and institutional barriers which would render SET problematic in both theoretical and practical contexts. Finally, we conclude by suggesting that if an ethical perspective is to be adopted we would steer away from normative ethical approaches and instead place an emphasis on other perspectives, such as ecofeminism.

## SET oriented marketing and virtue ethics

While not providing a detailed review of Virtue Ethics, the authors posit two key features that are particularly pertinent to how they have utilised the theory. First, it moves away from utilitarian arguments which focus on “more is better” and instead focuses on “enough is enough.” From a business/marketing perspective this “enough is enough” focus means it is unethical to both maximize profits and stimulate wants and desires. Such a perspective is obviously problematic to managerialists and marketers alike, indeed it could be argued that stimulating wants and desires is marketing’s raison d’être. Second, the authors argue that a reliance on virtue ethics focuses on flourishing communities whereby the primary focus is on the common good. Specifically they rely on an Aristotelian perspective by utilizing his four cardinal virtues of self-control, justice, practical wisdom, and courage as a means through which their concepts of “enough is enough” and community flourishing can be achieved. The authors offer us illustrations of how these four cardinal virtues can contribute towards SET.

We wonder why the more global perspectives so prevalent in the macromarketing and sustainability marketing critiques of marketing management have not be used by Dyck and Manchanda. Twelve years ago in earlier work that established a detailed ‘socio-ecological’ critique, Belz and Peattie (2009) managed to transform the 4 Ps to fit the purpose of ‘sustainability marketing’ and expounded how this needed to shift to a ‘sustainability marketing mix’ of customer solutions, communications, customer cost and convenience to have any chance of moving us towards a more sustainable economy. It must be noted that we believe even this work by Belz and Peattie falls short of the geopolitical challenge required for sustainability as it stands in 2021. Overall, however, it seems the extent of this detail from earlier work in marketing has been omitted from the SET analysis here. Furthermore, the way SET is framed, utilizes the inside perspective on sustainability which is very ‘firm centered’ and which is why the 4Ps as a toolkit becomes central to the authors’ key arguments. As such this analysis generates a weak form of sustainability focused on the firm and not a strong form of sustainability which places sustainability as the megatrend of our times (Kilbourne et al., 2018).

We argue, it is important for Dyck and Manchanda to take a more macro infused analysis of sustainability to enhance the explanatory power of how the ecosystem is effected by and damaged by market based systems. The authors miss the *The Theoretical Structure of Ecological Revolutions* (Merchant, 1987) which explains the foundational interconnectedness of ecology, so masterfully explicated by Carolyn Merchant, showing how Ecological Revolutions (major transformations in humans relations with nonhuman nature) altered New England. By taking this big picture view her work sought to explain how New England had been first transformed via an external colonial ecological revolution in the early seventeenth century and then again transformed in a capitalist ecological revolution, which took place roughly between the American Revolution and about 1860. It was here that Puritans, influenced by their interpretation of the Bible, legitimated agriculture and indeed sowed the seeds for present day market exchanges and practices of marketing management to have such strong effects. Merchant argued that the concepts most useful to understand environmental history are ecology, production, reproduction, and consciousness. She maintained that due to the differences in the immediacy of impact of production, reproduction, and consciousness on non-human nature, a structured, levelled framework of analysis is needed.

This framework provides a high level of appreciation of the ecosystem and the basis for an understanding of stability as well as evolutionary change and transformation. Although change may occur at any level, ecological revolutions are characterized by major alterations at all three levels. It is this overarching viewpoint that is lacking in Dyck and Manchanda’s work, but which other sustainability marketing researchers have at least acknowledged in existing works. As a result, the SET approach although well intended remains stuck between what is needed (sustainable consumption & production) and the vestiges of old tools from the past (the 4Ps) which marketers cling to! The complete rethinking on how to operationalize sustainability as *the* business imperative is not strong enough in this paper. As a consequence their analysis may again be ‘too little too late’ in a bid to rescue marketers away from a position of problem creators to one of ecological solution providers. These points are further developed below.

We critique the SET oriented marketing and virtue ethics approach outlined by Dyck and Manchanda as follows:

### Self-control

The authors argue that by applying principles of self-control, marketers will be able to focus on the bigger picture and not the self-interests of marketers or firms. This, we argue, is theoretically simplistic and practically maladroit. For example, it is not up to marketers individually to determine what the bigger picture is, this will be determined by a company’s overall strategy which in itself is a response to the much wider ecological system. More importantly, there has been a considerable literature, published over many decades which recognises the almost impossibility of determining wants from needs (Brownlie & Saren, 1992; Dixon, 1992; Houston, 1986). In their self-control example, Dyck and Manchanda argue for the development of products that “serve the larger whole” (p 6). Our counter argument to this would ask “what would this look like in reality,” “who gets to decide what the larger whole is,” and “how does one address potential conflicts between, say for instance, the larger whole of humanity versus the larger whole of the planet?” At the very least the role of marketing manager needs to expand to become ‘sustainability marketing manager’ and be in sync with other protagonists in the sector in which any firm operates.

### Justice

Once again it is not the job of marketers to consider those stakeholders who “lack voice or exist on the margins of society,” so this virtue is impossible to implement (at least by marketing managers). Similarly, how does one determine who is associated with a product/service and that they are treated fairly?

### Practical wisdom

Here, we circle back to the same argument, this is not the marketer’s job, and being aware of interconnectedness is meaningless if you are not in a position to do anything about it. A CEO is certainly better placed to act on awareness of ecological interconnectedness, but due to the socio-ecological and political combination in society (Prothero & McDonagh 2021) the challenge of sustainability cannot be extracted from politics. A CEO can instruct the company to operate on the principles of a closed loop system which appreciates the sustainability challenge as the megatrend of our times.

### Courage

Finally, being courageous from a socio-ecological perspective, is not in most marketing managers job description, and thus once again we argue this proposal is not applicable in a real world context. At the very least we would need to expand the role to that of a sustainability marketing manager where sustainability is central to the organization’s raison d’être. What if maintaining your integrity revolves around profit maximization? Who gets to decide? How does a marketing manager have the authority to go against the self-interests of the organisation they are employed by?

The arguments put forward by the authors of a need to develop both marketing theory and practice in such a way that social and ecological priorities take precedence over profit making are laudable, and we strongly support this view, as would Merchant’s tiered analysis of production, reproduction, and consciousness and Belz and Peattie’s sustainability marketing. However, we do not believe that SET marketing, as proposed by the authors, provides a means through which to achieve this. Overall, we argue that the SET framework is: (a) theoretically simplistic and practically maladroit; (b) while it is laudable to suggest marketers focus on a flourishing community, achieving this in organisations which focus on profit-making is unrealistic; (c) determining wants versus needs is problematic; (d) conflicts between social and ecological needs are ignored and the conflicts of both against over-riding economic priorities makes SET problematic; (e) interconnectedness is a central component of addressing social and ecological concerns, but this is not the primary aim of marketing managers within a business context; and at the same time, many marketing managers are not in a position to address such concerns nor do they have the requisite sustainability skillsets; and (f) much of the authors’ focus is on community and stakeholders, with nature taking a back seat! This is a verifiable anthropocentric focus which will not help solve the ecological crisis.

The use of the word *humankind* in the article’s title is problematic from a planetary preservation perspective. To continue to focus on human needs and a flourishing community or even convenience which is omitted here, although included by Belz and Peattie (2009), the analysis neglects the urgency for *restoration* of the ecosystem. This is particularly true when the ‘price of bio-diversity’ is not really analyzed as it is deemed too much of a challenge and that the literature has not considered it sufficiently. There is a literature on accounting for biodiversity which should be consulted when deliberating over this price issue (see Jones et al., 2013). For example, instead of talking about a flourishing community we need to urgently resolve how to mobilize governments and commerce to remove a landmass of plastic floating in the Pacific Ocean which is the size of France. This would be challenging to cost but not impossible and the manufacturers of plastic could be levied in the process for its removal and plastic ingredients more fairly ecologically priced as a result.

## SET oriented marketing and the marketing mix

As their SET approach is aimed at prioritizing social and ecological concerns we are perplexed by the authors use of the marketing mix as a means through which to demonstrate how SET might work in practice. The authors themselves note that the marketing mix has been criticized for many decades now (O’Malley & Patterson, 1998) and specifically for the sustainability challenge (Belz & Peattie, 2009). However, what is more pertinent in this context is that even if an SET approach could be implemented then it needs to be done so much sooner than at a marketing mix stage. The marketing mix are ‘old school’ tools and techniques through which to implement marketing strategies. Marketing strategies are determined as a result of overall business strategy. Thus, the initial emphasis has to come at the organisational and strategic level and not just the operational one. It does not matter how much courage a brand manager has if the marketing strategy is to sell more stuff through any means legally possible. We can provide illustrations of these problems through each of the SET examples offered by the authors. For instance:

**Table:** 

SET Oriented Marketing Mix	Limits of SET Analysis and related Concerns
Product – “refuses to offer unsustainable products that meet consumer wants but not their needs, even if such products are profitable (self-control).”	Who gets to distinguish between wants and needs? What geo-indicators are monitored to make this judgment—how would a marketing manager go against the wishes of their organization? Ecological evidence in relation to both sustainable consumption and production processes needs to be gathered and analysed across the organisation by a range of decision makers not just a marketing manager.
Price – “emphasizes that price is more than a mere financial transaction, it is also infused with relationships and involves a firm’s contribution to social justice and value creation (relational ethics, re-personalize price).”	What would this look like? How is it implementable? Would this lead to increased prices thus making the organization involved less competitive in the marketplace? As Belz and Peattie (2009, 23) note the customer cost does not only include the financial price a buyer has to pay for a product or service, it also considers the psychological, social and environmental costs of obtaining, using and disposing of a product. How is accounting for the restoration of biodiversity included in price calculations?
Place – “emphasizes place-based marketing channels that enhance socio-ecological well-being and local economies.”	How would this work in a globally inter-connected world? What about conflicts between social and economic well-being? How can we move local economy beyond a niche activity? What about supply of raw materials/products which are not available locally, but which have a global demand base, even for basic foodstuffs such as bananas?
Promotion – “promotes, describes, and offers (counter-cultural) alternatives that address negative socio-ecological externalities associated with the status quo.”	Who decides what counter-cultural alternatives are? How does one address negative socio-ecological externalities if these do not directly apply to the products the company is offering to the marketplace? For instance, such promotion needs to provide transparency with regards to the production process and access and disclosure of ecological evidence to support claims of operating sustainably. Such communication needs to coalesce around a geo-political mobilization to make sustainability *the* way of life (Kilbourne et al., 2018).

The use of virtue ethics as a means to tackle social and ecological ills through SET oriented marketing is, we believe problematic per se, and for many of the same reasons that FBL and TBL oriented marketing are equally problematic. That is not to say that we disagree with the importance of issues such as character and integrity in attempting to address these issues. We also recognise that a number of authors have attempted to consider virtue ethics from an environmental perspective (Sandler, 2007; Zwolinski & Schmidtz, 2013). However, we argue that in order to prioritize social and ecological issues above profit making means we must first look at much broader systemic and institutional issues that pervade the ‘logic of sustainability’ that has been appropriated in the marketplace (see Hill & McDonagh, 2020). Using the same theoretical mindset and its tools that got us into this situation to get us out of this emergency may not be the best use of our theoretical resources nor time.

Challenging the neo-liberal paradigm that most businesses thrive on means our challenging governments, business and society more generally as they interconnect to enable and support production and consumption. Indeed, as mentioned above there is a significant literature spanning many decades which considers these issues from both broad management and marketing perspectives. The authors themselves provide us with five key criterion required for SET marketing to become a world view, and some of these address our broad systemic and institutional concerns. However, what the authors fail to do is consider how such concerns render their SET approach as unworkable from a managerial perspective, and thus makes what they label five realistic outcomes, as incredibly unrealistic (at least on the scale required for them to have any meaningful ecological impact), and theoretically weak from an academic perspective.

## A more ecologically connected perspective to promote sustainability

Overall, in our opinion focusing on the sustainability marketing mix through a virtue based approach can only work if the bigger systemic and institutional issues are tackled in society more broadly. It becomes a geo-political project on a scale which surpasses collaboration which we have witnessed in attempts to quell the recent Covid-19, coronavirus pandemic. If we are to consider ethical approaches then we would argue for a steering away from normative ethical theories altogether and consideration towards more elaborate perspectives that are based on an interconnected appreciation of the system, one possibility is afforded by ecofeminism.

Ecofeminism has its roots in both feminist and environmental studies disciplines (d’Eaubonne, 1974; Merchant, 1987; Mies & Shiva, 1993) and has been discussed within the marketing discipline for a number of decades (McDonagh & Prothero, 1997; Dobscha & Ozanne, 2001; Littlefield, 2010; Dobscha & Prothero, 2012; Stevens et al., 2013; Maclaran & Stevens, 2019). Within marketing, these various authors have argued how an ecofeminist approach allows researchers to consider the systemic and institutional issues that are ignored when one only focuses on an operational tool such as the marketing mix. Maclaran and Stevens (2019, 243) argue that it allows us to question “taken for granted norms” and that:

“ecofeminism ultimately seeks to question binary systems that are socially constructed to devalue women and the environment, offering economic, spiritual and political reasons as to why patriarchal values are harmful and destructive, calling for an ethic of care and a transformational philosophy of a connected, human, ecological self, rather than an individualistic, androcentric, and anthropocentric self.” (Maclaran & Stevens, 2019, 239).

In a nutshell ecofeminism asks us to consider how a patriarchal system undermines both women and the natural environment, and the authors above have offered various suggestions as to how ecofeminist principles could contribute to addressing these problems within marketing. What they also do is recognise that it is the overarching ideology of neoliberalism which contributes significantly to both the climate crisis and a patriarchal society. In so doing, they differ significantly to the primarily operational SET and virtue-based approach proffered by Dyck and Manchanda. Ecofeminists address the systemic and the institutional head-on. They recognise that the climate crisis is borne out of an ideology focusing on competition and the free market and suggest both feminist and sustainability principles for addressing these taken for granted norms. These include focusing on inter-connectedness, collaboration and communal approaches, as also discussed in Dyck and Manchanda’s SET approach. Where they differ however is in their focus.

While it is beyond the scope of this commentary to discuss these at length we briefly raise ecofeminism here to stress there are longstanding schools of thought which do address the systemic and institutional perspectives missing in the Dyck and Manchanda article. Like the SET approach, we are not arguing here that an ecofeminist approach can solve the climate crisis and marketers role there-in overnight. What it does do though is offer enhanced depth of analysis and suggestions which could help us prioritize and tackle the systemic and institutional barriers to change, alongside operational strategies for marketers too. Such work would present sustainability as *the* political project of our times. We urge future marketing academics researching this topic to consider utilizing ecofeminist principles in their analysis and subsequent studies and actions.

## Conclusions

In conclusion, we would once again like to commend Dyck and Manchanda for their work, and for offering an alternative lens through which to explore social and ecological perspectives within marketing. This, in and of itself, is a laudable accomplishment, and such perspectives are still broadly absent from mainstream marketing outlets. We see the arguments put forward by the authors as being useful to stimulate the debate further. We also see potential for the article to be used in the classroom to discuss the application of ethical approaches to solving social/ecological approaches within marketing. That said, we offered a critique as to what we believe the theoretical and practical limits are within the article, and hope others consider these as they pursue research in the field in the future. Finally, we briefly introduce the reader to principles of ecofeminism and suggest such a perspective may offer more useful ways of thinking about the interconnections between production, reproduction and ecological consciousness, as well as how marketing and marketers become more cognizant of the politics of their role in contributing to a restorative ecology.
